# The Value of Labial Gland Biopsies as a Diagnostic Test for Sjögren’s Syndrome

**DOI:** 10.1007/s12105-024-01662-1

**Published:** 2024-07-03

**Authors:** Mollie Clark, Hannah Walsh, India Stephens-Laborde, Syed Ali Khurram

**Affiliations:** Unit of Oral and Maxillofacial Pathology, The School of Clinical Dentistry, University of Sheffield, Sheffield, S10 2TA UK

## Abstract

**Purpose:**

There are a number of diagnostic criteria that can be used to support a diagnosis of Sjögren’s syndrome (SS), a chronic autoimmune condition often characterised by xerostomia and xerophthalmia. Of the available investigations, the most invasive is the labial gland biopsy (LGB) for histopathology, which is associated with a risk of long-term altered sensation to the lip. A positive histological diagnosis is currently considered to be one of the most objective criteria, however there is debate about the interobserver agreement between pathologists, as well as the sensitivity and specificity of this test. We aim to determine if the diagnostic value of the LGB is significant enough to warrant the surgical procedure and its associated risks.

**Methods:**

This study involved assessing the degree of agreement between members of a pathology team for a cohort of 50 LGBs taken for the purpose of confirming or excluding SS. The Tarpley system was used, which involves the allocation of a ‘focus score’. Additionally, the histological diagnoses were compared to the relevant serological findings where available.

**Results:**

All cases within the cohort had adequate tissue for assessment. 84% agreement (Cohen’s Kappa = 0.585) was seen between the current team’s consensus and the original reporting pathologist on whether the appearance was supportive of SS. However, only 58% agreement was seen for focus scores (Weighted Kappa = 0.496). The agreement between the serology result and whether the histology was supportive of SS was 79% (Cohen’s Kappa = 0.493).

**Conclusion:**

The findings raise the possibility that undue emphasis is placed on the value of a histological SS diagnosis. The current system for assessing and grading these biopsies is ambiguous in nature, with a low threshold considered indicative of SS. Due to the risk of complications associated with a LGB, alternative minimally invasive investigations should always be considered. The histological findings in isolation, particularly when a low focus score is seen, may not be predictive of a diagnosis of SS.

## Introduction

Sjögren’s syndrome (SS) is a chronic autoimmune condition that can lead to reduced secretions from the exocrine glands of the mouth, eyes, genitals and skin. As a result, two of the most common clinical presentations are xerostomia and xerophthalmia [1]. The sequelae of this condition can include significant discomfort, secondary oral infections, periodontal disease, dental caries and altered vision. Furthermore, the diagnosis is associated with a 7–19 times increased risk of developing non-Hodgkin lymphoma [2]. SS can be categorised as either primary disease occurring on its own, or arising secondarily to other autoimmune conditions such as rheumatoid arthritis and systemic lupus erythematosus.

According to the American-European Consensus Group (AECG) for Sjögren’s Syndrome associated with Johns Hopkins Sjögren’s Centre, there are six criteria, including signs, symptoms and positive investigation findings, that are considered supportive of a diagnosis of primary SS (Table 1). The presence of at least four of the six criteria, which must include positive histopathology or serum autoantibodies, is sufficient for a diagnosis of SS [3]. Alternatively, any three of the four objective criteria can also confirm the diagnosis. A histological diagnosis is achieved using a labial gland biopsy (LGB), which involves the surgical removal of minor salivary glands from the lower lip. The minimum criteria for a positive result is defined by the AECG as focal lymphocytic sialadenitis, which is one focus of more than 50 lymphocytes per 4mm^2^ of normal glandular tissue.

**Table 1:** Summarised AECG diagnostic criteria for Sjögren’s syndrome

	Criteria	Objective/Subjective
I	Ocular symptoms	Subjective
II	Oral symptoms	Subjective
III	Ocular signs (including an abnormal Schirmer’s test)	Objective
IV	Histopathology of a labial gland biopsy showing focal lymphocytic sialadenitis	Objective
V	Oral signs (including low unstimulated salivary flow or abnormal sialography)	Objective
VI	Serum autoantibodies (anti-SSA (Ro) and/or anti-SSB (La))	Objective

Typically, on receipt of a LGB, the tissue is fixed in formalin and stained with haematoxylin and eosin for microscopic analysis. The most frequently used grading system for evaluating LGBs is the Tarpley system (Table 2) [4]. First proposed in 1974, the Tarpley system does not allude to a minimum amount of tissue for a diagnostic LGB. However, it is generally accepted that tissue comprising at least 4 good sized minor salivary gland lobules, or a minimum of 8mm^2^, must be submitted for analysis [5]. In practice, the scores outlined by the Tarpley system are allocated based on analysis of a representative area of glandular tissue measuring 4mm^2^, in accordance with the AECG guidelines [3]. Of note, Tarpley et al. state that: **“**the lip biopsy must be regarded as an adjunctive parameter rather than the sine qua non for diagnosis” [4].

**Table 2:** The Tarpley system of histological grading for labial gland biopsies

Score	Definition
0	Normal or mild non-focal sialadenitis
1+	1 or 2 aggregates of at least 50 mononuclear cells
2+	More than 3 aggregates of at least 50 mononuclear cells
3+	Diffuse mononuclear infiltration with partial acinar destruction, with or without fibrosis
4+	Diffuse mononuclear infiltration with or without fibrosis destroying the lobular architecture completely

Of the investigations for SS, the LGB is by far the most invasive as it involves a surgical procedure to remove some minor salivary glands from the lower lip. This procedure is associated with a number of risks, including long term impaired sensation at the biopsy site [6]. Given these risks, the importance and usefulness of a histological diagnosis for SS should be examined.

The other, less invasive, objective investigations for SS are serology for relevant autoantibodies and sialography. The serological autoantibodies of interest typically include anti-nuclear antigen (ANA), rheumatoid factor (RF), and anti-extractable nuclear antigens (ENA), including anti-SSA (Ro) and anti-SSB (La). Anti-SSA (Ro) is regarded as the most common autoantibody in SS, with a frequency of 33–74%, followed by anti-SSB (La) with a frequency of 23–52% [7]. Sialography, the technique of radiographic imaging following the injection of contrast medium into a salivary duct, is a low-risk procedure causing minimal discomfort to the patient. When performed and assessed by an experienced radiologist, sialography has been shown to have diagnostic accuracy over 90% for SS [8]. Furthermore, although it does not currently feature in the AECG diagnostic criteria (Table 1), simple ultrasonography is increasingly used as a non-invasive diagnostic technique, with evidence of high sensitivity and specificity.

## Methods

In this study, we aimed to assess if the diagnostic value of LGBs is significant enough to warrant the surgical procedure and the associated risks. Histopathology and oral medicine clinical records were used to retrospectively identify patients who had undergone a LGB for the purpose of confirming or excluding SS, as well as the histological diagnosis, relevant blood test results and follow-up data. There was no direct patient involvement in this study, and all identifiable information was anonymised at the point of data collection.

The study cohort included all 50 patients with available records who had undergone a LGB at the Charles Clifford Dental Hospital in Sheffield, UK. These records span 5-years, between 2012 and 2017. Of these 50 patients, 43 had available records of relevant blood tests, and 30 had follow-up data. The serological value selected for analysis was the ‘extractable nuclear antibody’ (ENA) result, which was deemed positive if the presence of anti-SSA (Ro) and anti-SSB (La) antibodies were detected.

All slides were screened to ensure that a minimum of 4 lobules of minor salivary gland tissue were present to obtain a diagnosis. Blind analysis of the histological slides was completed by a Consultant in Oral and Maxillofacial Pathology as well as two speciality trainees. For each case, the team determined a consensus for the focus score according to the Tarpley system of grading, which the involved clinicians had practical experience of. It was also recorded if the team considered the histological features supportive of a SS diagnosis, irrespective of whether the score met the previously defined threshold of 1+.

All data was inputted into Microsoft Excel (2016) and stored in an anonymised-linked format. The statistical tests used were Cohen’s Kappa for the binary categorical variables (report conclusion and serology), and Weighted Kappa for the ordinal categorical variable (focus score).

## Results

All 50 of the LGBs included at least 4 lobules (> 8mm^2^) of minor salivary gland tissue and were therefore all considered adequate for assessment. Furthermore, all original pathology reports referred to a focus score, in line with the Tarpley system of grading. Of the 50 cases, 43 had records of relevant blood test results, including an ENA, completed at similar time to the biopsy.

The first variable that was examined was the agreement between the original reports and the current team consensus on the binary variable of whether the histology supports a SS diagnosis. None of the current team were involved in the original diagnoses. 84% agreement was seen (Cohen’s Kappa = 0.585), with 42/50 cases remaining unchanged in their conclusion. 18% were unanimously deemed supportive of SS, whereas 66% were not considered in-keeping with the disease. It is notable that 16% of cases were concluded differently by the current team, which highlights that the examination of these specimens is subjective and should not be considered definitive in isolation.

**Fig. 1:**
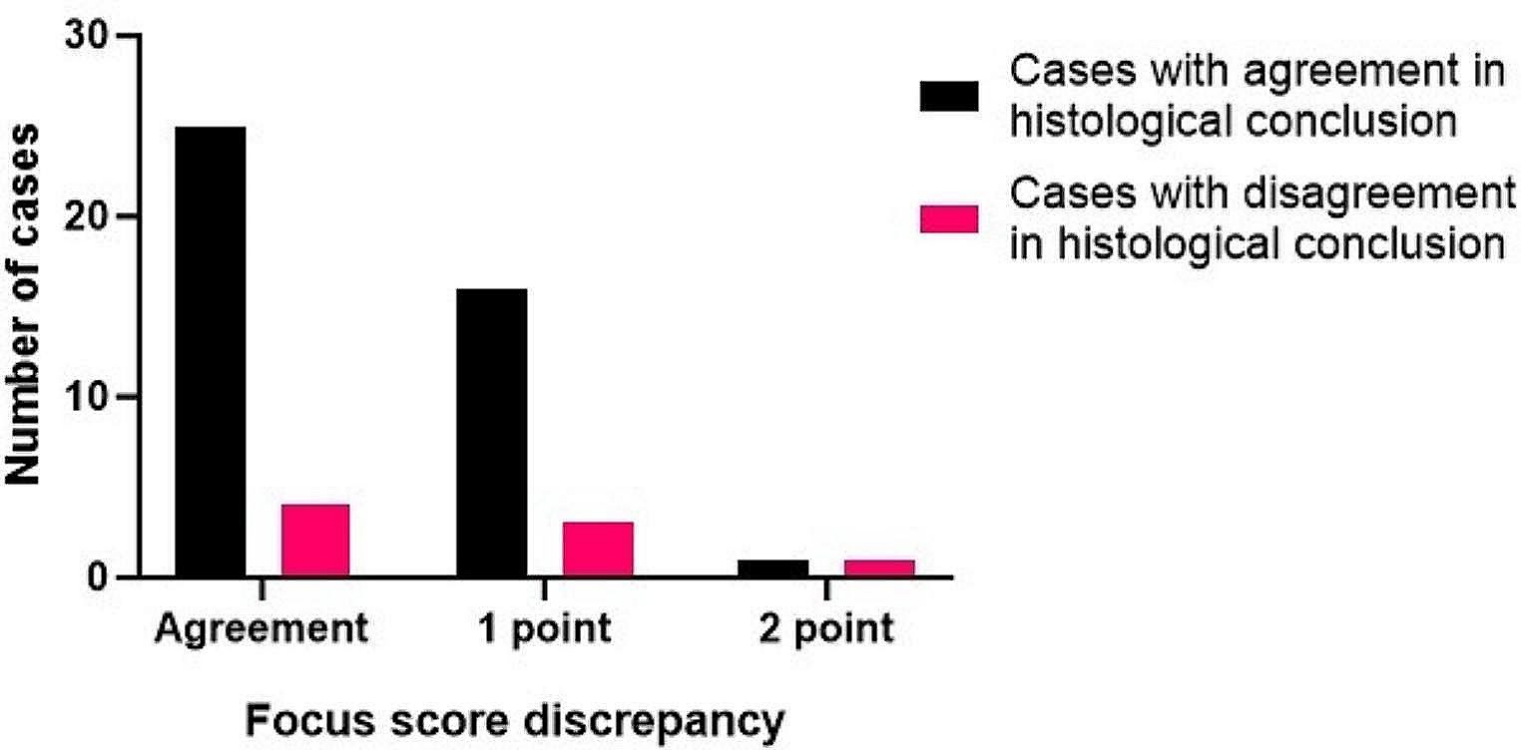
The cases with agreement or discrepancy between the consensus opinion of the current team and the original pathology report on focus scores. Separated by the current team consensus on agreement with the original report conclusion

A lower degree of agreement was seen when examining the consensus on the Tarpley system score allocated to each biopsy. 58% complete agreement was seen, with disagreement of more than one point observed for 2 out of the 50 cases (Weighted Kappa = 0.496) (Fig. 1). The disagreement was largely seen between the scores ‘0’ and ‘1+’, again highlighting the subjectivity of these scores.

Notably, as highlighted by Fig. 1, there were several cases where agreement was seen on the allocated focus score but there was disagreement on whether this appearance was supportive of a diagnosis of SS. This reflects the flaws of the current grading system and the potential overlap of SS with non-specific inflammation of the minor glands.

**Fig. 2:**
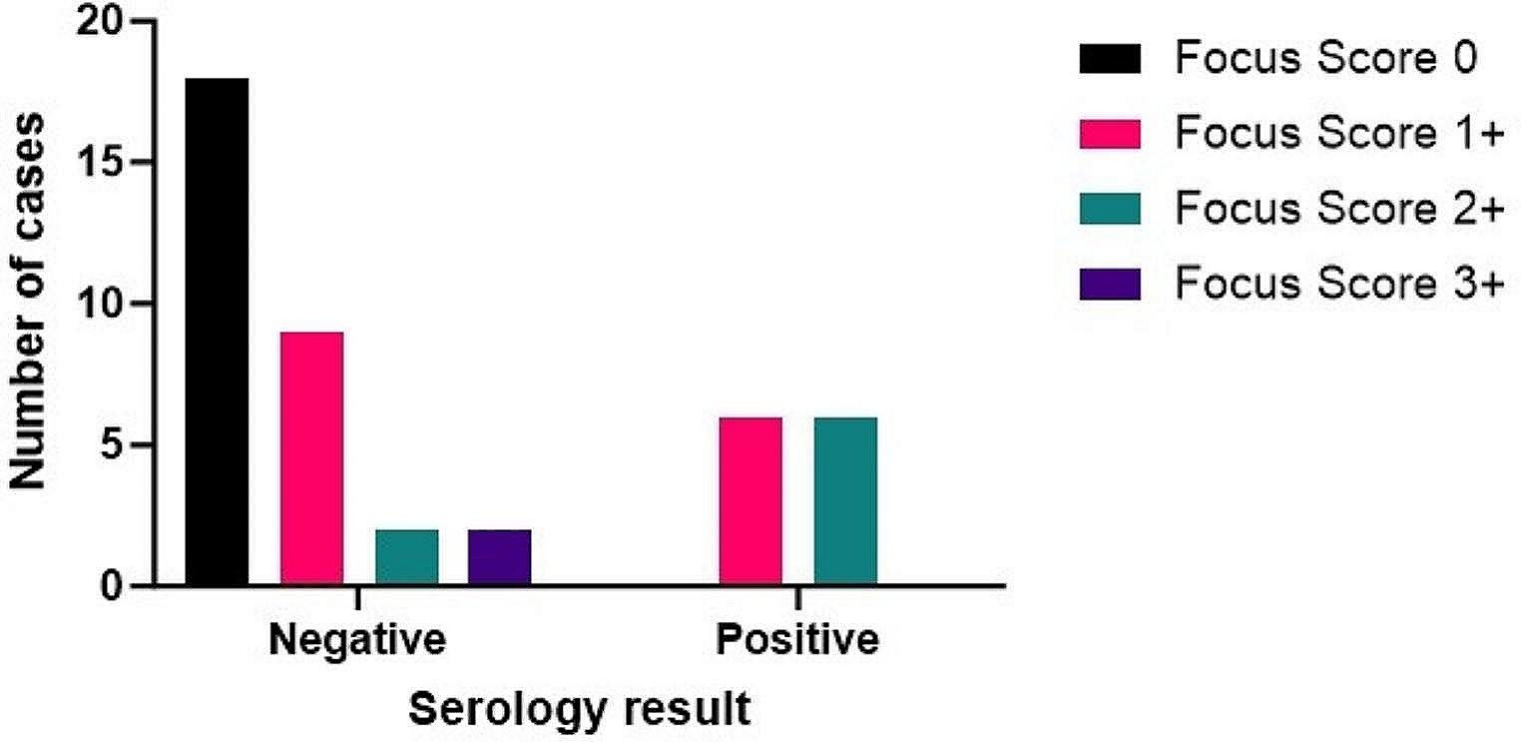
The distribution of focus scores allocated by the current team within both positive and negative blood test result categories

The association between the original reports and the relevant blood test results was also analysed. 79% of cases had ENE serology which correlated with the conclusion of the original pathology report (Cohen’s Kappa = 0.493). It can be seen in Fig. 2 that there were no cases with both positive serology and a focus score of 0, as determined by the current team. However, there were a high number of cases with focal lymphocytic sialadenitis, including 2 cases with a focus score of 3+, which did not have blood test results supportive of SS. This may reflect the lack of specificity of histological findings in SS.

Of note, cases where biopsy was performed despite positive blood results make up 28% of the cohort. It should be considered that, when four of the criteria outlined in Table 1 are present, both positive histology and positive serology are not required for a definitive diagnosis of SS. Arguably, all minimally invasive investigations, such as blood tests and sialometry, should be exhausted before a LGB is considered as an option, as this may put the patient at an unjustifiable risk.

Finally, the rate of post-operative complications following LGB within the cohort was examined. Clinical records of follow-up appointments could be found for 30 of the patients. In 53% of cases, no post-operative complications were record. 27% of patients experienced numbness of the biopsy site and surrounding areas, while 20% described paraesthesia. Persistent scarring causing discomfort was noted in just 1 case. Of note, the mean duration of follow-up was just 3.4 months, often as a result of discharge following negative findings. Further dedicated studies may be warranted to determine the incidence and duration of post-operative complications for LGBs.

## Discussion

**Fig. 3:**
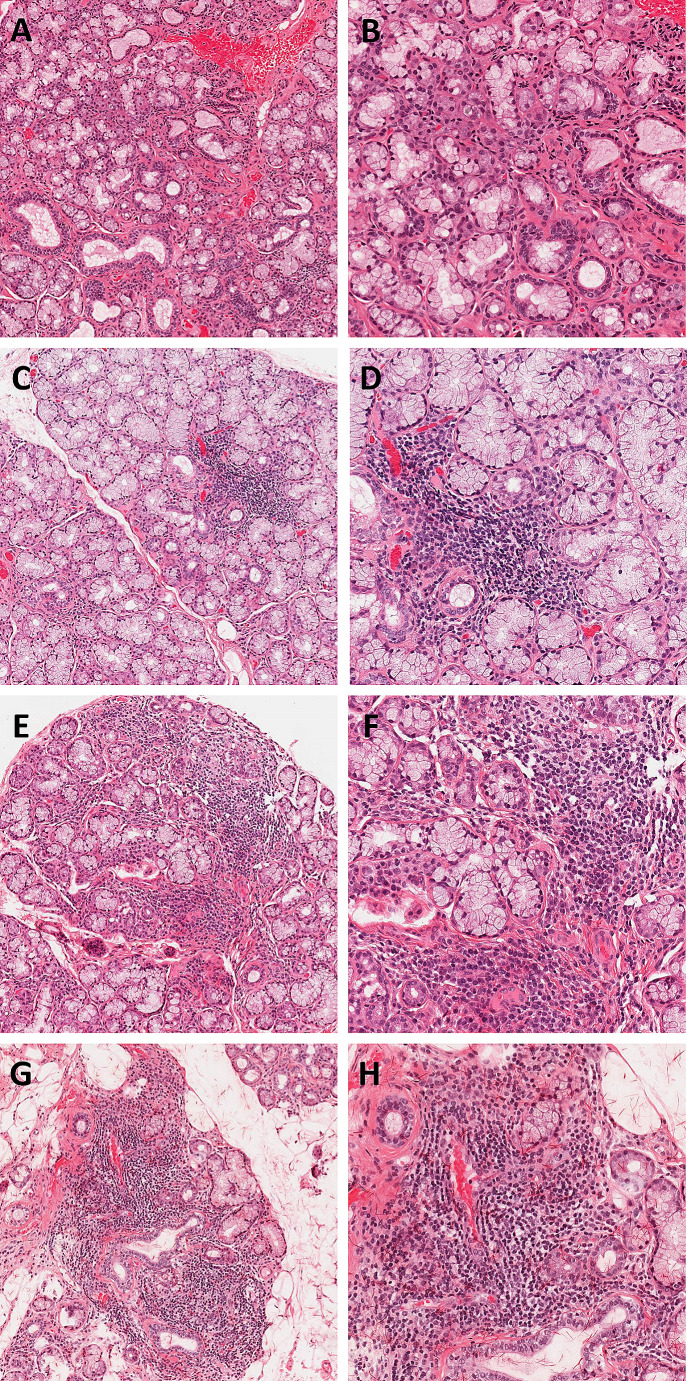
Examples of labial gland biopsy (LGB) histology. (**A**) LGB focus score 0, 10x magnification; (**B**) LGB focus score 0, 20x magnification; (**C**) LGB focus score 1+, 10x magnification; (**D**) LGB focus score 1+, 20x magnification; (**E**) LGB focus score 2+, 10x magnification; (**F**) LGB focus score 2+, 20x magnification; (**G**) LGB focus score 3+, 10x magnification; (**H**) LGB focus score 3+, 20x magnification

**Fig. 4:**
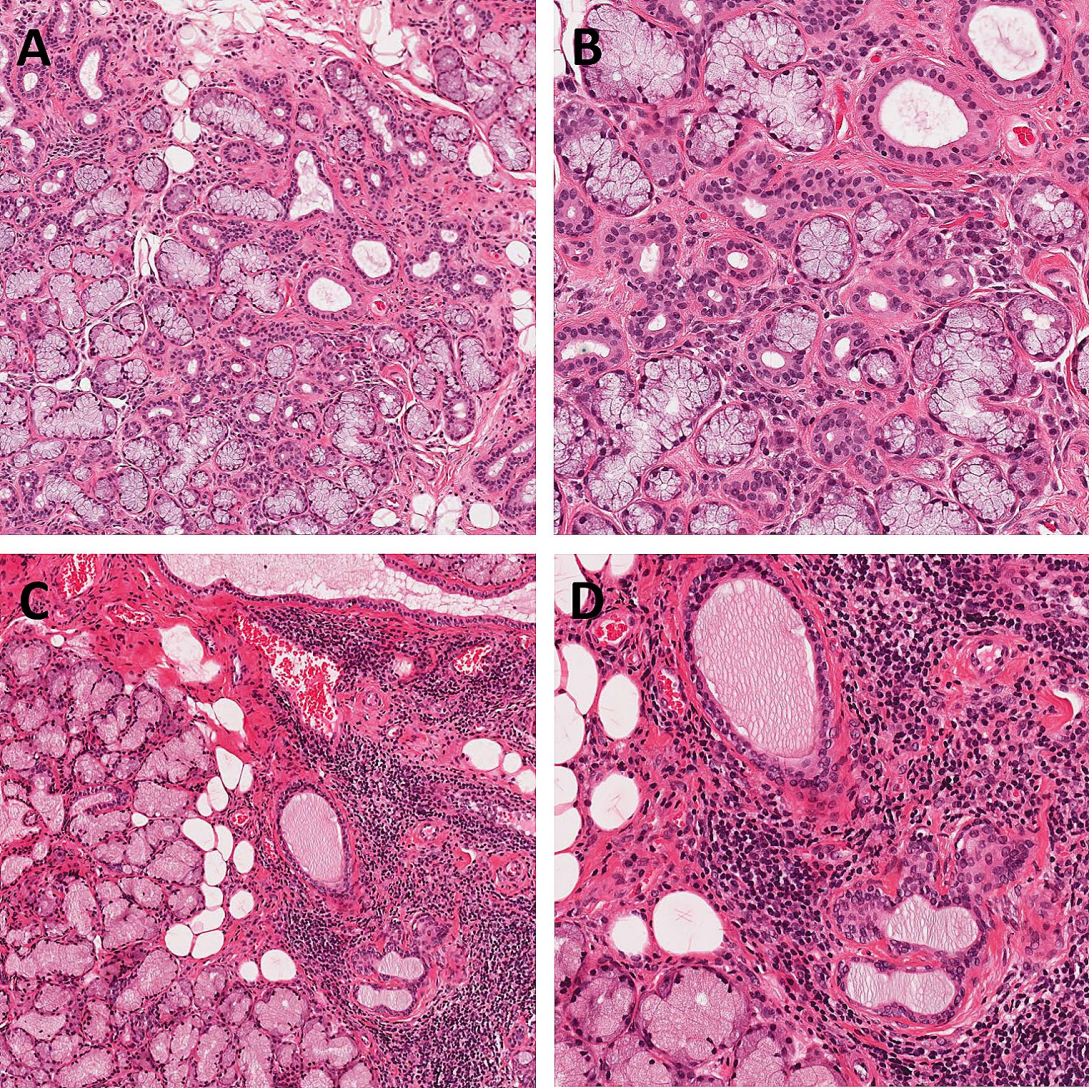
(A) False negative LGB where histology showed a focus score of 0 despite positive serology and ultrasound, 10x magnification; (B) False negative LGB, 20x magnification; (C) False positive LGB where histology showed a focus score of 1 + despite negative serology and ultrasound, 10x magnification; (D) False positive LGB, 20x magnification

In the development of their proposed grading system, Tarpley et al. found a correlation between a higher focus score and the detection of ‘serum anti-salivary duct antibody’. However, within the 96 patient cohort, there was little inclusion of healthy patients or patients with non-immunologically mediated salivary gland disease [4]. One of the key histological mimics of SS related-focal lymphocytic sialadenitis is non-autoimmune sialadenitis, which often has an infective or traumatic aetiology. It can be particularly challenging to distinguish these entities on small LGB specimens, as both may display aggregates of chronic inflammatory cells, as well as fibrosis and acinar destruction. Furthermore, all these findings have been shown to increase in prevalence in both major and minor glands as patient age increases, and have been described as physiological age-related changes [9, 10]. This must be considered as a potential confounding factor in the assessment of LGBs, as the prevalence of SS increases 5-fold in patients over the age of 65 years [11]. Figure 3 shows examples of LGBs with a range of focus scores from the cohort examined within this study. Figure 4 shows examples of false positive and false negative LGB histology, highlighting the diagnostic challenge these cases can present.

A systematic review conducted by Guellec et al. in 2013 examined the predictive value of LGBs in primary SS. This review defined a positive LGB as having a focus score of >1, in line with the AECG diagnostic criteria (Table 1). They found that the sensitivity of LGBs ranged from 63.5 to 93.7%, while the specificity ranged from 61.2 to 100% [12]. The authors comment on limitations of this diagnostic test and the need for revaluation of the existing guidelines, which is supported by the findings of this study.

When discussing the diagnostic value of LGBs, the risks to the patient must also be considered. A 2022 reported that 21% of 630 patients who had undergone a LGB described long term impaired sensation (> 6 months), with 32% expressing that this affected their quality of life [6]. However, the rates of complication vary widely across the literature. Another study from 2008 reported the frequency of persistent paraesthesia 6-months after a LGB as 0.2% for a cohort of 502 patients [13]. The variability in reported rates of occurrence of complications may be a result of differing biopsy and investigation techniques. Clinician experience, patient recall and anatomical variation are also likely to impact the findings.

Regardless of the true prevalence of complications, it is essential that clinicians seek to arrive at an accurate diagnosis with minimal risk to the patient. Although not currently included in the diagnostic criteria outlined in Table 1, ultrasonography of the major glands is increasingly utilised as a reproducible and non-invasive alternative to both parotid sialography and LGB in assessing salivary gland disease. A 2013 study found a highly significant correlation between histological findings and ultrasound results for both patients with SS and those with non-specific sialadenitis [8]. A further study in 2015 determined that ultrasound scans of the major salivary glands had an impressive negative predictive value of 96%, as well as a positive predictive value of 85% [14]. These findings indicate that the disease process is likely to be relatively consistent in all minor and major salivary glands.

Additionally, there have been more recent advances in non-invasive investigations for SS. A number of detectable salivary biomarkers have been found to have a strong association with the disease, although further evaluation of clinical applicability is needed [15]. The development of investigations for more specific salivary and serum biomarkers, combined with pre-existing minimally invasive tests, could negate the need for an invasive biopsy in many patients.

This study was limited by a relatively small cohort of 50 cases from a period of 5 years. As a result, many of these cases will have been reported by a small group of pathologists, albeit different from the current team. Further studies spanning greater time periods and geographic locations may provide more representative data. Secondly, the only non-histological variable that was examined was the presence or absence of a supportive blood result. Patients with suspected SS will often undergo a variety of investigations, including blood tests, ultrasound sialography and sialometry, as well as thorough clinical examinations by multiple teams. Future work that compares all these findings may be useful is assessing their relative value.

## Conclusion

This study has raised the possibility that undue emphasis is placed on the value of LGBs in supporting a SS diagnosis. The sensitivity of this investigation should not be overestimated, as our current team consensus suggested that 16% of the cases examined would have been concluded differently from the original pathology reports. We believe the current system for assessing and scoring these biopsies is too ambiguous in nature with a low threshold, i.e. just one focus of lymphocytes per 4mm^2^ considered indicative of SS. A more appropriate system may be one that introduces more detail, guidance and categories, with scores that represent the presence of lymphocytic sialadenitis in the absence of definitive evidence for SS.

With the risk of complications associated with a LGB, alternative investigations should always be considered. It is good practice to exhaust all minimally invasive techniques, like sialography, serology and ultrasonography, before resorting to an invasive and potentially harmful biopsy.

## Data Availability

Not applicable.

## Code Availability

No datasets were generated or analysed during the current study.
